# Policy options to facilitate cancer genomic variant data sharing: outcomes of a modified policy Delphi

**DOI:** 10.1093/jlb/lsad022

**Published:** 2023-07-14

**Authors:** Jill O Robinson, Amira Daoud, Janis Geary, Vasiliki Rahimzadeh, Juli Bollinger, Christi J Guerrini, Robert Cook-Deegan, Amy L McGuire, Mary A Majumder

**Affiliations:** Center for Medical Ethics and Health Policy, Baylor College of Medicine, Houston, TX, 77030, USA; Center for Medical Ethics and Health Policy, Baylor College of Medicine, Houston, TX, 77030, USA; Consortium for Science, Policy & Outcomes, Arizona State University, Washington, DC, 20006, USA; Center for Medical Ethics and Health Policy, Baylor College of Medicine, Houston, TX, 77030, USA; Center for Medical Ethics and Health Policy, Baylor College of Medicine, Houston, TX, 77030, USA; Berman Institute of Bioethics, Johns Hopkins University, Baltimore, MD, 21205, USA; Center for Medical Ethics and Health Policy, Baylor College of Medicine, Houston, TX, 77030, USA; Consortium for Science, Policy & Outcomes, Arizona State University, Washington, DC, 20006, USA; Center for Medical Ethics and Health Policy, Baylor College of Medicine, Houston, TX, 77030, USA; Center for Medical Ethics and Health Policy, Baylor College of Medicine, Houston, TX, 77030, USA

**Keywords:** cancer, data sharing, genomics, knowledge commons, policy Delphi, precision oncology

## Abstract

Sharing cancer gene variant and relevant clinical data could accelerate progress in cancer genomics. However, data sharing is currently impeded by issues related to financial sustainability, equity, incentives, privacy and security, and data quality. Evidence-based policy options to facilitate data sharing in these domains, and ultimately improve interpretation of cancer-associated genomic variants, are therefore needed. We conducted a modified policy Delphi with expert stakeholders that involved generating, evaluating, and ranking potential policy options to address these issues, with a focus on the US context. We found policy options in the financial sustainability domain were highly ranked, particularly stable funding for trusted entities. However, some Delphi panelists noted that the culture of public research funding has favored short-term grants. Panelists favored policy options focused on action by funders, which had the highest overall total scores that combined effectiveness and feasibility ratings and priority ranking within domains. Panelists also endorsed some policy options connected to actors such as journals, but they were more skeptical of policy options connected to legislative actors and data resources. These findings are critical inputs for policy makers as they consider policies to enable sharing of cancer gene variant data to improve health.

## I. INTRODUCTION

Cancer genomics, or precision oncology, is a subfield of precision medicine dedicated to understanding the genomic bases of tumor cell proliferation and cancer-causing mutations. Secure sharing and use of cancer gene variant and related health data drive innovation in precision oncology because they maximize the potential for accurate variant interpretation necessary for prevention, diagnosis, and treatment. Research funding agencies in the US and globally have thus implemented policies that condition funding for cancer research on sharing the resultant data.^1^ National genomics initiatives are also heavily invested in large-scale repositories, which pool data and enable sharing.^2^

Building a knowledge commons is a prominent model to ameliorate many of these impediments, as the model prioritizes research collaboration over proprietary exclusion and community stewardship over institution-based data control. Data commons, or knowledge commons, are designed for broad use to accelerate discovery.^3^ Further, in cancer genomics, the community of data contributors and end users includes clinical laboratories and other health care providers.^4^ Creating a cancer variant data commons has, for example, been a key strategy for characterizing structural *BRCA1/2* variants^5^ known to increase breast and ovarian cancer risk, achieving sample sizes needed to conduct correlative analyses involving rare pediatric cancers,^6^ and helping to refine machine learning strategies for accurately predicting cancer variant pathogenicity in diverse patient populations. Formidable practical, ethical, and policy issues currently limit the transformative potential of an interoperable commons for sharing cancer gene variant data. Concerns include precarious funding, a lack of data standards, burdens associated with sharing, limited ancestral diversity in existing data sets, and data privacy and security. While, as noted above, some large-scale research-oriented data repositories enjoy significant support, other data resources, such as those that bridge the research and clinical realms or are smaller in scale or more targeted, struggle to secure stable funding.^7^

In addition, data structures remain inconsistent across repositories. Many of the largest cancer gene variant databases lack common standards for data capture, variant interpretation, and knowledge representation. These issues impose burdens on those sharing data, reduce usefulness of the data, and impede researchers and clinicians.^8^

Preparing data for sharing requires significant time and effort. Yet historically researchers have rarely been compensated for the time and costs needed to share data, unless explicitly funded to create a new data resource or repository. Data cleaning, archiving, annotation, and depositing are secondary to primary research goals and therefore infrequently built into grant budgets, despite requirements to comply with data-sharing policies. The rapid pace of variant data generation looms as another challenge. The ability to generate DNA sequence data currently exceeds the analytic capacities of many clinical laboratories, hindering the interpretation of variants and creating a bottleneck^9^ with critical consequences for the future of molecular diagnostics needed to deliver precision oncology care. Moreover, clinical variant reporting still requires manual oversight and often *de novo* curation,^10^ despite automation and advances in cloud computing that enable informaticians to compute larger and more complex variant datasets than ever before. To understand the scale of this problem, one study found that of 48,036 cancer patients treated at one institution over a 6-year period, 28.6 per cent of clinically reported variants were not already present in public variant resources. This increased the number of new variants requiring curation from 1.72 to 3.73 variants per assay.^11^ The authors note that the scalability problem will be heightened as more patient genomes (and larger regions of their genomes) are sequenced, and they call for ‘a set of global genomic variant knowledgebases’ to ‘reduce the duplication of curation effort across laboratories’ among which data are often not shared.^12^

Further, due to the lack of ancestral diversity in existing data repositories, new variants of uncertain significance are more prevalent and difficult to interpret in groups with predominantly non-European ancestry who are underrepresented in research and often have limited access to clinical genetic testing.^13^ This problem only exacerbates existing health inequities.

Also, the scientific utility of the data commons rests on adequate phenotypic and genomic data collection, and on linking records between clinical care and research.^14^ Responsible and sustainable approaches to data sharing must be connected to specific data type(s), but different types of data and data access structures present different risks and challenges.^15^ For example, proof-of-concept studies show that individuals may be re-identified when functional genomic data are shared, so sharing these data without adequate disclosure of the risks could compromise trust and willingness to contribute other types of data that sustain the commons.^16^ Furthermore, individuals may have more concerns about genomic data sharing when those data can be accessed by researchers based in other countries,^17^ or by commercial entities.^18^ Difficulties with international data sharing compound as more data are migrated to cloud-based storage and automated systems enable researchers to securely access data anytime, from anywhere, but where applicable data protection laws can be nebulous.^19^ Legal constraints and institutional policies have led some scholars to question whether data protection laws and institutional incentives are doing more to constrain than enable global data commons in genomics.^20^

Although there are recognized obstacles to contributing, accessing, and using shared data, a persuasive case has been made that increasing sharing of cancer gene variant and related data will promote health.^21^ Yet, there is currently no set of stakeholder-vetted policy options for expanding and improving the cancer variant data commons. Policy options need to be developed that anticipate the future needs of such resources as the trajectory of precision oncology research and care evolve.

We previously reported on issues identified and prioritized though a modified policy Delphi process, an iterative process using various approaches (eg surveys, interviews, meetings) with expert stakeholders to address a specific policy question.^22^ The highest-priority domains were financial sustainability, equity, incentives, privacy and security, and data quality. In this paper, we present the outcomes of the final round of the modified policy Delphi process to identify effective and feasible policy options to address these issues, along with related points to consider. While our focus here is on the US context, as is most evident in the domains of incentives and privacy and security, we recognize that supporting cancer genomic data sharing is a global enterprise and have highlighted international dimensions in our other work.^23^

## II. METHODS

### II.A. Study Design and Population

One of the goals of a policy Delphi is to identify underlying pros and cons of a proposed policy option, rather than reaching consensus as in a traditional Delphi. Policy Delphi panelists can be informed stakeholders and those primarily impacted by the policy under study and need not be technical experts. In our policy Delphi, panelists fulfilled one or more of the following roles: data contributor/end user (patients, families, advocacy organizations); data generator (testing laboratories); data source (databases); data facilitator (data resources, curators, annotators, variant interpreters); professional data user (genetic counselors and other clinicians, researchers); and policy scholar (Table 1). Our modified approach included interviews, facilitated discussion at a virtual meeting, and surveys with Delphi panelists.^24^

**Table 1:** Characteristics of Delphi panelists

	*N* = 22	%
Gender		
Female	13	59
Male	9	41
Age		
30–39	6	27
40–49	7	32
50–59	7	32
60–69	1	4.5
Missing	1	4.5
Location		
US	18	82
International^*^	4	18
Cancer commons role^†^^,^^**^		
Policy experts/scholars	16	73
Professional data users	6	27
Data contributor/end users	4	18
Other	4	18
Data sources	3	14
Data facilitators	2	9
Data generators	1	4.5

^*^International includes Canada (*n* = 3) and UK (*n* = 1).

^†^ *N* = >22 (100%) as participants could identify with more than one category.

^**^Data generators = testing laboratories; data contributors/end users = patients, families, advocacy organizations (both patient and non-patient advocacy); data sources = databases; data facilitators = data resources, curators, annotators, variant interpreters; professional data users = genetic counselors, clinicians, researchers.

We previously reported on the first three rounds of the Delphi process.^25^ In the first round, we conducted interviews with twenty-four expert stakeholders comprising our Delphi panel to elicit policy issues they perceived as impeding data sharing and the development and sustainability of a cancer gene variant commons. We then administered a survey to rank and rate the policy issues previously identified. In the third round, we hosted a virtual meeting with twenty-two Delphi panelists to generate potential policy options responsive to the highest ranked issues according to the survey results. Separately, we interviewed 24 domain experts who were not part of our Delphi panel,^26^ identified from snowball sampling and a review of the literature, to generate additional potential policy options and pro/con arguments.

This paper focuses on the fourth and final round of the Delphi process, which consisted of a survey to rate and rank the policy options synthesized from the facilitated discussions in the third round, and the additional interviews conducted with domain experts. Seventy-three policy options were generated across five key policy domains (summary of issue): ‘incentives’ (some entities that generate data are not sharing data because of countervailing incentives and values), ‘financial sustainability’ (the commons has characteristics of a public good, which makes ensuring long-term sustainability challenging), ‘privacy and security’ (trust in the security of a commons is difficult to build given that privacy breaches can never be completely eliminated, protections vary by jurisdiction, and laws/regulations/norms protecting privacy change over time. Further, links among data sets are needed to interpret cancer risk, but then the data becomes more identifiable and privacy risks increase), ‘equity’ (the commons should not perpetuate existing inequities in health care, or create new ones. Uses should aim to address inequalities), and ‘data quality’ (data are of variable quality and there is no consensus about how to monitor and assess quality). ^27^ To reduce respondent burden, some options were removed based on consensus of the study team after considering how often panelists and interviewees suggested the policy option, as well as the strength of consensus in support of the option. The final number of policy options presented to panelists was 52 (seven to 13 options per domain) (Supplement Table 1). The survey was pre-tested with members of our research team who were not involved in survey development and finalized.

The survey asked panelists to rate the effectiveness and feasibility of the policy options for each domain using a four-point Likert-type scale ranging from Strongly Disagree to Strongly Agree, without a neutral midpoint. A ‘Don’t Know’ response option was included, although per policy Delphi best practices, panelists were encouraged to use it sparingly.^28^ After completing the rating tasks in each domain, panelists were presented with the full list of policy options for that domain and asked to rank their top three policy options from highest to lowest priority. The survey suggested actors who could, in theory, implement policy options within each domain. These actors, broadly defined, included data resources, funders, health insurers, journals, professional societies, proficiency testing programs, public-private partnerships, and ‘others,’ which were defined to include researchers, institutions, companies, and end users of data. Specific examples of actors were provided at the beginning of the survey, and panelists were instructed to provide any details and/or identify other relevant actors in open-ended responses after completing the rating and ranking questions in each domain. The survey is reproduced in the Supplementary Materials.

Delphi panelists were emailed the link to the final survey (administered via Qualtrics online survey platform) and received up to three email reminders. Panelists were offered $100 compensation to complete the survey. Surveys were administered between May 4 and June 1, 2022.

All study materials were approved by the Baylor College of Medicine Institutional Review Board (protocol H-46047).

### II.B. Data Analysis

For each policy option, we used Excel to create weighted scores for ratings and rankings (Supplement Table 1). Effectiveness and feasibility scores used the following weights: Strongly Disagree = −2, Disagree = −1, Agree = +1, Strongly Agree = +2. For example, in response to whether the first policy option listed in the incentive domain (I1: ‘Health insurers could exclude labs from preferred networks if they don’t share’) was effective, one panelist strongly disagreed, three disagreed, seven agreed, and nine strongly agreed; the effectiveness score was therefore twenty. The highest possible score for all policy options for each of effectiveness and feasibility was 44 and the lowest possible score was −44.^29^ We also calculated the percentage who selected the Don’t Know response for the effectiveness or feasibility rating of any policy option. A priority rank score was created for each option by multiplying the number of times panelists ranked an option in the top three in terms of priority, weighted as follows: if an option was ranked ‘1’ (highest priority) it was weighted as three, if ranked ‘2’ it was weighted as two, and if ranked ‘3’ it was weighted as one. We then summed across the effectiveness, feasibility, and priority rank scores to generate an overall total score to determine the top options with the highest scores within a domain.

Although we were primarily interested in these overall total scores to determine the top options within a domain, we also wanted to compare policy options by actor type across domains. Priority rank scores are not directly comparable across domains as the score depends on the number of options within a domain (ranging from seven options for financial sustainability to 13 for data quality). We therefore normalized scores to generate a priority rank ratio by dividing the Priority Rank Score by the Total score possible over the number of options within the policy option domain.

We grouped policy options based on the actor identified in the option to compare the average effectiveness, feasibility, and priority rank ratios by actor type. Each policy option was connected to an actor, with the exception of two policy options in the data quality domain. Supplement Table 1 shows the actor-policy option assignments.

Because panelists’ decision-making rationales are important outcomes of the Delphi process,^30^ we analyzed the open-ended responses of Delphi panelists’ reasons for their ratings and rankings along with the quantitative data. We first organized the responses by the pros and cons and other points to consider for the policy options within each domain. This helped us to better understand why some policy options were rated and ranked higher than others. Next, two members of the research team (AD, MAM) independently coded the responses for themes to understand the points to consider and to identify supporting illustrative quotes for the top three policy options (based on overall total score) in each domain. Research team members then met to reach a consensus on the themes and the corresponding illustrative quotes.^31^

## III. RESULTS

All but two Delphi panelists completed the survey (*n* = 22). Panelists reported multiple roles related to a cancer genomic variant commons, with the majority reporting some relevant policy expertise (eg a biomedical researcher who has become very active in helping to develop data-sharing policies) (Table 1).

We present the domains in order of Delphi panelists’ overall total score from highest to lowest scored domains: financial sustainability, equity, data quality, incentives, and privacy and security (Supplement Table 1). Although more nuanced than straightforward, this order could indicate which domains have more feasible, effective, and higher priority policy options to pursue. For example, policy options in the financial sustainability domain had the highest average feasibility score (16.9) and third highest average effectiveness score (16.1), while equity had the highest average effectiveness score (17.1) and second highest feasibility score (14.8) compared to other domains (Supplement Table 2).

### III.A. Results by Actor across Domains

Across all domains, Delphi panelists rated and ranked funders and journals as the actors connected to options that were most strongly endorsed (Table 2). One open-ended response indicated that mapping to actors with the power to motivate researchers to share would ensure data sharing: ‘Funders and journals have the best sticks for ensuring the sharing of research data.’ (ID 017) Another panelist noted, however, ‘We’re asking a lot of funders, and yet we don’t have a stable source of funding.’ (ID 022) One policy option under data quality listed professional societies as developers of standards; that option was rated highly in terms of effectiveness and feasibility but was not ranked as a high priority option to pursue. Indeed, panelists commented that professional societies might not ‘have the requisite expertise for standards development’ or ability to enforce standards once developed. (ID 022) As one panelist noted, ‘even if ACMG [the American College of Medical Genetics and Genomics] makes a statement or recommendation, it isn’t actually a “law”.’ (ID 019) Actors connected to options least often, as endorsed across policy domains, included private companies, private–public partnerships, and the US Congress. Notably, policy options connected to the US Congress were rated as effective but not feasible, with at least one panelist explaining why: ‘Congress is dysfunctional’ (ID 015).

**Table 2:** Top three policy options by domain and actor connected to issue statement

		Actor^*^			
Domain	Issue statement	Funders	Journals	Data resources	Other^**^
Financial sustainability	Stable funding (FS1)	✔			
	Broaden existing resources (FS7)	✔			
	Low-cost storage (FS4)	✔			
Equity	Support small studies if with underrepresented (E6)	✔			
	Equip lower-resourced groups to use data (E8)	X		✔	
	Community needs assessments (E1)	✔			X
Data quality	Data-sharing infrastructure (Q2)	✔			
	Quality checks (Q8)			✔	
	Comply with standards (Q1)	✔			
Incentives	Publication conditional on data sharing (I9)	✔	✔		
	Peer-review data-sharing plans (I4)	✔			
	Monitor for and punish noncompliance (I5)	✔			
Privacy and security	Federated models (PS10)	✔			✔
	Novel technologies (PS9)	✔			✔
	Transparency about risks and harms (PS8)			✔	✔

^*^Cells marked with an X indicate that the actor was identified as connected to a policy option in a panelist comment rather than in the framing of the policy option. Checkmarks indicate that the actor was included in the framing of the policy option.

^**^Other includes: clinical labs (PS10), individual researchers (E1, PS10, and PS8), institutions (PS10, PS9, and PS8), and end users of data (PS10, PS9).

### III.B. Results by Domain

In the sections below, we present quantitative ratings and rankings, as well as points to consider from participants’ open-ended responses for the top three options (based on overall total score) for each domain. A table with illustrative responses for each point to consider is included for each domain. Tables and figures for the quantitative data can be found in supplementary materials. Since the total score is an integrated measure, it does not convey divergences across measures of effectiveness, feasibility, and priority rank. Higher priority rank generally aligned with higher effectiveness and feasibility rank scores, as shown in Supplement Table 1 and the Supplement Figures. However, in a few instances effectiveness and feasibility scores diverged to the point that one was negative and the other was positive. For example, as we note below, policy options requiring action by Congress had negative feasibility scores even with positive effectiveness scores, which may have contributed to lower priority rank.

#### III.B.1. Financial Sustainability

To address the issue of financial sustainability, panelists prioritized options that were connected to funders as actors. Overall, the option for funders to provide stable funding for trusted entities, like the Global Alliance for Genomics and Health (GA4GH) (FS1), had the highest total score. The option of funders broadening use of existing data resources (eg ClinVar) (FS7) had the second highest total score, and the option of funders investing in low-cost data archiving/storage (FS4) was third.

Funders providing stable funding for trusted entities (FS1) was also rated as most effective. However, panelists commented that this option would require a culture shift and questioned the grounds for determining that an entity is trusted (Table 3), which may explain its lower feasibility ratings relative to the other two top options. Investing in low-cost data storage (FS7) was viewed as feasible and pragmatic but concerns about its effectiveness were noted, especially if this led to storage of data that lack value. Finally, broadening the use of existing data resources (eg ClinVar) (FS4) was rated as highly feasible; panelists noted that tapping into existing resources would be responsible and minimize waste. Several panelists commented that combining options to broaden and merge existing resources (FS7 and FS3) could also be effective.

**Table 3:** Financial sustainability top policy options

Issue statement	The commons has characteristics of a public good, which makes ensuring long-term sustainability challenging	
Top three policy options (label)^*^	Points to consider	Illustrative quotes (study ID)
Funders could provide stable funding for trusted entities (eg GA4GH) (FS1)	Builds on proven track record	`Prioritizing funding of GA4GH IMHO has incredible value if funding and resources were to expand driver projects like BRCA Exchange' (013)
	Define ‘trusted’	‘I would need to know what the criteria for “trust” is, and who makes these criteria. For whom is GA4GH a trusted partner? What is the ground of trust?’ (004)
	Requires a culture shift	‘Stable public fund commitments seem in principle like the most sustainable option … [T]he culture of public research funding tends to prioritize relatively short-term research grants. A shift in this culture would probably be required if sustainable funding for resources such as a cancer commons were to become a feasible option’ (009)
Funders could focus on broadening use of existing data resources (FS7)	Responsible choice	‘Rather than building new resources from scratch, it seems more feasible and responsible to build on existing resources and support trusted entities with a strong track record’ (016)
	Minimizes waste from reinvention and unproductive competition	‘There is a tendency to constantly be reinventing new platforms/resources, that only add to the competition for limited resources’ (024)
Funders could invest in low-cost data archiving/storing (FS4)	Viewed as pragmatic and equitable	‘Taking pragmatic steps like low-cost storage…are feasible and effective as well as equitable’ (012)
	Need to be selective to not waste resources	‘Not all data are valuable and so low-quality archiving is a waste of resources’ (017)

^*^See supplementary tables for all policy options labeled by domain (number), eg FS1 for the first list policy option in the financial sustainability domain.

Notably, options in the financial sustainability domain connected to actors other than funders, namely data resources (FS6) and public–private partnerships (FS5), were not highly rated or ranked (Supplement Figure 1). Private financing of data resources via fee-for-service models (ie pay-to-play or involving access fees) (FS6) was one of only two options rated negatively for effectiveness in the survey. Panelists commented that these models are exclusionary, have a poor track record of supporting data resources, and perpetuate inequities.

#### III.B.2. Equity

The top overall and most feasible policy option to address equity issues required funders to create mechanisms to support studies with small sample sizes from underrepresented populations (E6, Supplement Table 1). At the same time, panelists expressed concerns about the limited impact of small studies and statistical power, although one noted that data could be aggregated across small (and larger) studies to mitigate analysis issues (Table 3). These concerns likely explain the lower priority rank for this option compared to the other top options in this domain, despite its high effectiveness and feasibility scores (Supplement Figure 2). Notably, to some extent panelists rated each option in this domain as effective and feasible, with some commenting that they were ‘mostly in support of all these options’ to address equity (ID 004).

The second top overall policy option to address equity issues, and the only top option not connected to funders in this domain, was data resources equipping lower-resourced institutions/communities to utilize data to conduct research (E8). This option aligned with calls for greater transparency and increasing awareness by fostering communication (Table 4).

**Table 4:** Equity top policy options

Issue statement	The commons should not perpetuate existing inequities in health care or create new ones. Uses should aim to address inequities.	
Top three policy options (label)^*^	Points to consider	Illustrative quotes (study ID)
Funders could create funding mechanisms to support studies with small samples if from underrepresented populations (E6)	Recognize the role of statistical power in creating inequities	‘It is almost impossible to build well-powered studies with most of the subjects coming from underrepresented populations. Which prevents such studies from happening and thus creates inequity. Something about this equation has to change!’ (010)
	Aggregation of samples as a response to concerns about statistical power	‘Small sample size funding would be great only if those small sample were married (federated, integrated) with larger databases to provide greater power for analysis’ (012)
Data resources could equip lower-resourced institutions/communities to utilize data to conduct research (E8)	Alignment with targeted funding streams and greater transparency	‘I like the pairing of funding for specific studies by institutions focused on under-represented populations along with greater transparency about data diversity for studies which don’t fit into those buckets’ (006)
	Fostering communication leads to increasing awareness	‘[Supporting low-resourced institutions from underserved communities to engage in their own research is among the options that] foster communication between patient communities and researchers, which increase transparency, and increase awareness within the patient communities on how the research may benefit their communities’ (014)
Funders could conduct community needs assessments to identify community priorities and create greater alignment between funding and those priorities (E1)	Who is best positioned to carry this out?	‘Researchers, not funders, are probably best places to align scientific and community aims (through engagement)’ (024)
	Relationship building is key	`[R]elationship building *is* something that’s been effective in fostering greater equity in research. A fine example of that is the policies of the Alaskan Native Tribal Research Consortium, which support[s] greater inclusion of tribal representation throughout the research process' (014)
	May be well intentioned but could potentially be counter-productive	`The key to equity of outcomes should be equity in governance, by including and empowering all those who contribute to the resources, particularly those whose bodies and lives are the source of data. Arrangements which treat data subjects as ``others'' to be ``engaged” or subjected to ``community needs assessment'', however well-intentioned, perpetuate inequity. Serious thought therefore needs to be given to how to build properly equitable governance arrangements, with real decision-making powers over how the resource should be used, for what purposes and to whose benefit' (009)

^*^See supplementary tables for all policy options labeled by domain(number), eg E1 for the first list policy option in the equity domain.

The policy option with the third highest overall total score was funders conducting needs assessments to align funding and community priorities (E1). Panelists’ lower effectiveness and feasibility ratings for this option aligned with the questions they raised about who should conduct these assessments and whether they are sometimes counterproductive and ‘perpetuate inequity’ if undertaken in lieu of giving ‘those whose bodies and lives are the source of data’ a meaningful role in governance (Table 4) (ID 009).

#### III.B.3. Data Quality

Funders supporting the data-sharing infrastructure (eg setting standards, data cleaning, and curation) (Q2) was the top overall option to address data quality, rated as the most effective and feasible and ranked as the highest priority option (Supplement Table 1). Nearly a third of panelists (6 of 22) commented about the importance of funders supporting data-sharing infrastructures rather than simply mandating data sharing. Panelists noted, however, that a plan for developing and enforcing standards is not easy to implement, particularly without incentives to abide by those standards (Table 5). Thus, the third overall top option addressed funders incentivizing data contributors to comply with standards, such as giving those who comply with standards preferred access to data resources or funding their access to data resources (Q1).

**Table 5:** Data quality top policy options

Issue statement	Shared data are of variable quality and there is no consensus about how to monitor and assess its quality.	
Top three policy options (label)^*^	Points to consider	Illustrative quotes (study ID)
Funders could fund data-sharing infrastructures (eg setting standards, data cleaning, curation) (Q2)	Value of data cleaning and curation	‘fund curation, clap clap clap clap clap’ (015)
	Enforcement of standards is important, but also difficult	‘Biocuration is one of the biggest needs out there! Some of it goes back to problems with clinical workflows: that data is often not collected in a form that’s amenable to downstream analysis. The first thing that could change the picture [is] enforced standards for data quality, coupled with support for the biocuration activities that are often needed for ensuring data quality’ (014)
	Prioritize data quality over data sharing	`[W]e’re asking a lot of funders, and yet we don’t have a stable source of funding. Do we prioritize other aspects of making data available and let recipients deal with data quality issues? Not sure I would trade quality improvement at the data access point for some of the other aspects of data sharing for which funding is needed...' (022)
	Not as simple as you think	‘[D]ata quality and standards are always relative to intended use, and standardization inevitably constrains what can be done with a resource. Moreover, in favoring some uses over others, standardization also favors some actors over others. Before thinking about standard-setting, you therefore need to think about what a resource is for and [who] should benefit from it’ (009)
Data resources could include rigorous quality checks in data selection and curation processes (eg gnomAD) and/or attach quality ratings by standard metrics to data (eg ClinVar) (Q8)	Gatekeeping incentivizes generation of higher-quality data	‘In general, I believe in the effectiveness of policies that enforce the quality of data being ingested over those that mark the quality of data already ingested, because the former pushes the submitters to generate better-quality data’ (014)
	Requires resources	‘[W]here are the resources for that – and to what extent are standards recognized that would enable this kind of check?’ (022)
Funders could incentivize data contributors to comply with standards (ie preferred access or funding access to data resources) (Q1)	Incentives for data contribution are the key to success	‘Incentives are needed to get anyone to do this work. Standards also help the work, once incentivized, to get done well’ (012)

^*^See supplementary tables for all policy options labeled by domain (number), eg Q1 for the first list policy option in the data quality domain.

The second overall top option was the only one not connected to funders. It addressed data resources including quality checks in data selection and curation, and/or adding data quality ratings based on standard metrics (Q8). This option was rated lowest in feasibility compared to the other top three overall options (Supplement Figure 3). In comments, some panelists questioned the existence of rigorous standards, as well as what resources would be used to support them (Table 5).

Panelists did not support the option for data resources to crowdsource data characterization (ie describing the quality of the data) while also providing attribution criteria for deposited data (Q11) or the option for data resources to implement artificial intelligence (AI) approaches to clean, extrapolate and interpret clinical significance of variant data (Q9). Neither of these policy options was ranked in the top three by any participant, and Q11 was rated negatively for effectiveness and feasibility, while Q9 was rated negatively for feasibility. In the comments, one participant noted that crowdsourcing data characterization would heighten privacy issues, while others raised concerns about biases and inequities in AI.

#### III.B.4. Incentives

Journals requiring sufficient data for replication to publish (I9) had the highest overall total score for this domain, reflecting one panelist’s comment that journals have the best ‘sticks’ for ensuring the sharing of research data. (ID 017) However, concerns about how complete the data would need to be and difficulty with ensuring compliance, expressed in some panelists’ comments, may have impacted feasibility ratings and priority rank scores. Funders were connected to the other top two options within the incentives domain (Supplement Table 1). Panelists commented that requiring peer review of data-sharing plans at the research funding application stage (I4) would be easy to implement because some grants already require this (Table 6). However, other panelists noted that criteria for such reviews can be ambiguous, and evidence of impact is low. Perhaps for these reasons, this option had the lowest effectiveness rating of the three top options (Supplement Figure 4). Funders monitoring compliance with approved plans and sanctioning noncompliance by withholding funds or future funding (I5) was rated as effective and was the highest ranked option in terms of priority, but concerns about the costs of monitoring impacted feasibility ratings. Additionally, one panelist noted potential negative impacts on investigators’ careers given that the end of funding may not align with the timing of completion of the work.

**Table 6:** Incentives top policy options

Issue statement	Some entities that generate data are not sharing data because of countervailing incentives and values	
Top three policy options (label)^*^	Points to consider	Illustrative quotes (study ID)
Journals could condition publication on submission of sufficient data for replication (I9)	Enforces an existing best practice, but addresses questions of scope	‘Submitting data for replication of studies should be standard...though there are issues of how complete those data can or should be’ (010)
	Assessing compliance on a case-by-case basis may limit impact	‘Journal and funder constraints could work but may not have as big of an impact because it would only be on a case-by-case basis’ (006)
Funders could require that data-sharing plans be peer-reviewed for grant selection (I4)	Easy to implement	‘Some grants already require data sharing plans, so this is easy to pursue’ (016)
	Review criteria are unclear	‘Just having a data-sharing plan is not likely to be as effective because anyone can draw one up for grant submission purposes … Peer review of data sharing plans is an interesting idea - but I don’t know there is sufficient expertise out there to appropriately judge them’ (022)
	Experience to-date raises questions about impact	‘Depending on NIH review of plans has proven completely ineffectual so far, and there is no reason to think that it will improve in the future’ (001)
Funders could monitor funded projects and withhold funds or future grants from applicants who do not comply with approved data-sharing plans (I5)	Potential for harms to investigators	‘The problem is that there can be a long lag between the end of funding of a grant and actual completion of the work under a grant. A withholding of funding clause could then put an investigator out of business simply because they had not yet finished their research plan’ (010)
	Monitoring is expensive	‘Merely asking for data plans does not mean they will be implemented. Monitoring is effective but costly’ (011)
	Post hoc efforts are less effective/feasible	‘In my experience, post hoc monitoring/incentives [are] much less effective/feasible than getting folks to share from the get-go’ (012)

^*^See supplementary tables for all policy options labeled by domain (number), eg I1 for the first list policy option in the incentives domain.

Panelists were less supportive of policy options connected to health insurers and data resources. Policy options connected to health insurers (I2 and I3) raised concerns about negatively affecting or burdening patients. One panelist commented that making data sharing a checkbox for laboratories could encourage ‘share-washing,’ meaning the sharing of unhelpful data for compliance purposes (such as submitting variant classifications to ClinVar without metadata or supporting evidence) (ID 014). Panelists also raised concerns about data resources using tiered access (I6) and providing compensation to data contributors (I7) due to the harms associated with limiting access by or otherwise penalizing those who are not in a position to share their data or do not generate data to share.

#### III.B.5. Privacy and Security

Having a range of actors, including funders, contribute to the adoption of federated models (PS10) was the top overall option regarding privacy and data security, rated as the most effective policy option. It also ranked highest in priority for this domain (Supplement Table 1). Federated models were viewed as protecting privacy due to less need for data duplication. Slightly lower feasibility ratings likely reflected concerns about utility compared to costs and questions about evidence of success of these models (Table 7).

**Table 7:** Privacy and security top policy options

Issue statement	Trust in the security of a commons is difficult to build given that privacy breaches can never be completely eliminated, protections vary by jurisdiction, and laws/regulations/norms protecting privacy change over time. Further, links among data sets are needed to interpret cancer risk, but then the data becomes more identifiable and privacy risks increase.	
Top three policy options (label)^*^	Points to consider	Illustrative quotes (study ID)
Funders, clinical labs, individual researchers, institutions, and end users of data could adopt federated models of data sharing to avoid having a centralized database, where data are uploaded and downloaded locally, which would minimize the risks of re-identification and reduce harms from security breaches (PS10)	Fewer copies = greater security	‘The best improvement on data protection is to have fewer copies of the data requiring protection. Federation is a great step forward for this’ (014)
	Need to evaluate tradeoffs (utility)	‘Federated models do reduce the risk - but at what cost to utility (hence why I said, “don’t know” from a feasibility standpoint)’ (022)
	Need evidence that federated models work in hereditary cancer context	‘Federated models have a part to play (privacy by design), but risk being interpreted too narrowly (data never moves) and have still not been shown to be feasible outside some health surveillance networks’ (024)
Funders, institutions, and end users of data could invest in the development and use of novel technologies geared toward protecting privacy and enhancing data security (eg leveraging synthetic data to reduce re-identification risk, and leveraging secure computational methods to allow analysis of data without moving data (PS9)	Prevention is best	‘Technological measures to prevent data breaches/misuse are the best method of preventing these occurrences’ (001)
	Technology is still immature	‘Privacy-preserving technologies have a part to play but remain immature …’ (024)
	Need to recognize ‘arms race’ dynamic with bad actors	‘While I think that investing in the development and use of novel technologies geared toward protecting privacy and enhancing data security is a good idea, it is an arms race with no end in sight unless there is lawmaking that removes the incentives to hacking these data’ (012)
	Need to weigh tradeoffs (utility)	‘Technical safeguards (eg differential privacy, homomorphic encryption, secure multiparty computation, federated learning) can offer measurable, effective privacy protection but they have a cost in terms of computational efficiency, accuracy of results, and administrative overhead’ (021)
	Faux ideology of effective technology	‘[E]veryone wants magic tech to fix this, but I haven’t seen any of that tech work at anything approaching meaningful scale’ (015)
Data resources, institutions and individual researchers could be more transparent about security risks and potential harms (PS8)	Transparency may lead to public pressure on lawmakers	‘Research on privacy risks and costs will help spur lawmaking as will greater transparency (and the public awareness that comes with it)’ (012)
	Need balanced, non-alarmist messaging	‘Along with the idea of being more transparent about risks and harms, it is important also to mention benefits and gains’ (010)
	Transparency alone is not a solution	‘Just being more transparent/clear about the risks seems to do little to solve the problem’ (022)

^*^See supplementary tables for all policy options labeled by domain (number), eg PS1 for the first list policy option in the privacy and security domain.

Having a range of actors, including funders, invest in, develop, and use novel technologies (eg synthetic data, secure computational methods) (PS9) was rated as slightly less effective and slightly more feasible than PS10 (Supplement Figure 5). Four panelists commented that technological advances are the best methods for preventing data breaches and misuse (Table 7). At the same time, panelists noted that these technologies are immature, might expose data security vulnerabilities, and have utility tradeoffs ‘in terms of computational efficiency, accuracy of results, and administrative overhead’ (ID 021).

The final top option by total score in this domain was data resources, institutions, and individual researchers being more transparent about security risks and potential harms (PS8). Panelists noted that this option could raise awareness and ‘spur lawmaking’ but would need to be balanced with information about potential benefits and would not effectively address privacy and security by itself (ID 012).

Policy options connected to the US Congress and data resources were rated negatively for feasibility. Five panelists strongly agreed that policy options connected to Congress (PS1–PS3) would be effective policy options, while strongly disagreeing that they would be feasible (Supplement Figure 5). Eleven panelists commented about the need for congressional action but lacked faith that Congress would follow through on implementation. Another policy option that panelists ranked low for feasibility was creating a mechanism for data resources to compensate those whose privacy has been violated by security breaches (PS5). Five panelists commented that data resources compensating for privacy violations due to security breaches would be inadvisable, because it would entail figuring out the dollar value associated with each security breach. As one participant noted, ‘compensation of individuals seems like a bottomless pit’ (ID 001).

## IV. DISCUSSION

In the final solutions-focused round of our Delphi process, 11 of the 15 top policy options were mapped to funders,^32^ such as the National Institutes of Health (NIH) and private foundations. The range of funder actions included providing stable funding for data resources and related initiatives like GA4GH, supporting specific efforts to advance equity and implementation of standards related to data quality, strengthening incentives for data sharing, and safeguarding privacy, as summarized in Table 2.

Panelists noted that movement forward on the top overall policy option to provide stable funding would require a culture shift, as both public and private funders perceive their role as investing in innovative ideas at their inception rather than providing long-term, stable funding. Funders may have an expectation that once a data resource or initiative is established, users will take over funding. However, plans to charge researchers fees for downloading from the widely used Genotype-Tissue Expression dataset were abandoned,^33^ and clinical laboratories have not embraced pay-for-access data resources like BRCA Share™.^34^ Notably, panelists largely rejected pay-for-access models. Funders’ emphasis on innovation creates challenges for established data resources, even though a case can be made that such resources catalyze a tremendous amount of third-party innovation. Finally, data resources like ClinVar have both research and clinical applications, and research funders may see clinician support as outside their purview. Examples of widely used resources with long-term NIH funding (typically through the National Library of Medicine’s National Center for Biotechnology Information) exist, including GenBank and the Genome Reference Consortium. Still, the precariousness of resource funding is recognized as a problem in the NIH’s Strategic Plan for Data Science, which notes that historically the agency ‘has often supported data *resources* using funding approaches designed for *research* projects.’^35^ The Plan commits to adopting funding approaches for databases and knowledgebases that are appropriate for resources and focused on metrics such as user service, utility, interoperability, and efficiency. It also calls for recognition of the role of the NIH in a larger data-science ecosystem that includes health care providers and extends to related initiatives to develop and support adoption of standards and tools. This is progress, but our panelists’ responses suggest that leaders in the NIH Office of Data Science Strategy, and data science champions at other funding agencies, still face resistance in implementing this vision.

Several panelists expressed support for a ‘pull all levers’ approach by funders to address equity issues. The option that scored highest overall was funding studies with small sample sizes from underrepresented populations. In comments, panelists highlighted the tension with standards related to statistical power. Like others who have noted these kinds of concerns as impediments to advancing equity, they emphasized the importance of engaging in creative problem-solving rather than simply accepting a status quo that leads to inequities.^36^ Also, given that the focus is often on underrepresented racial and ethnic minority groups, it is important that funders avoid unintentionally adopting a reified view of race or ignoring complexities related to ancestry. A committee of the National Academies of Sciences, Engineering, and Medicine recently published a report addressing challenges in the use of race and ethnicity and other population descriptors in the genetics and genomics research context and setting out best practices.^37^ These and other aspects of equity are addressed at greater length and with more nuances in Geary et al.^38^

If financial sustainability is addressed, this is likely to bring about progress in other domains. With long-term, stable funding, groups like GA4GH would be well-positioned to continue to support progress on data quality and privacy and security. For example, in the privacy and security domain, our panelists favored adoption of federated models for data sharing, but one panelist had concerns about feasibility. GA4GH has invested considerable effort in championing federated models, making the case for their adoption, and demonstrating the feasibility and utility of a federated approach through one of its driver projects, BRCA Exchange.^39^

Regarding incentives, a new NIH-wide Data Management and Sharing Policy took effect in January 2023. Some commentators published articles or submitted comments to the NIH calling for strong data-sharing requirements in line with our panelists’ preferred policy options, such as peer review of data management and sharing plans as part of the grant selection process.^40^ The NIH rejected these calls in the final Policy.^41^ The NIH emphasized the need for flexibility given ‘the substantial variety in research fields, projects, and data types’ and justified reliance on program staff for review in terms of the benefit of consistency. However, a study of implementation of the National Cancer Institute’s Cancer Moonshot Public Access and Data Sharing Policy found that a similarly flexible approach was not effective when applicants did not fully understand the importance or details of the policy. The researchers concluded that ‘reliance on applicant-generated plans, rather than concrete agency mandates, could result in plans that overlook or undervalue data sharing.’^42^

As for actors other than funders, panelists were leery of options that put the sole onus for action on data resources. Their comments explained that they wanted to protect data resources from being saddled with too many demands, especially given financial sustainability concerns. That said, panelists did see an important role for data resources in several domains, as shown in Table 7. For example, they identified a role for data resources in equipping lower-resourced institutions to use data to conduct research. Some promising NIH- or National Science Foundation-supported efforts focused on helping data resources play this role include the Genomic Data Science Community Network^43^ (with links to the National Human Genome Research Institute Analysis, Visualization, and Informatics Lab-space data resource) and the IndigiData workshop^44^ (with links to the Native BioData Consortium data resource). Panelists also saw a role for data resources in ensuring transparent yet balanced communication about privacy risks and addressing quality either through metadata and quality ratings or through gatekeeping to ensure that only high-quality data enter the commons.

Finally, panelists recognized a role for journals in providing incentives for researchers to share data. To date, journal policies have been imperfect tools for increasing data sharing, but the difficulties have led to proposals for improvement.^45^ A key recommendation in a recent paper is incorporating a strong evaluation component in journal policies to ensure that data are indeed shared, and then implementing penalties for noncompliance with data-sharing commitments (eg embargoes on future publications from authors who have not complied with commitments in connection with prior publications).^46^ However, it may be unrealistic to expect journals to police meaningful compliance given the time and resources required. As a more moderate step, journals could emulate the ‘PLOS Genetics’ policy that asks reviewers and editors to consider the functional utility of the planned approach to data sharing when evaluating the potential impact of a manuscript.^47^

What does this leave out? The clinical realm. Our Delphi panelists focused on policy options that would mainly augment the efforts of researchers generating and using data. Of course, sharing of research data has significant benefits in advancing understanding and treatment of hereditary cancer. Even if we focus on clinical applications of the genomic knowledge commons, resources like gnomAD that aggregate data from research laboratories have utility in variant classification in the clinical context, as set out in the American College of Medical Genetics and Genomics standards and guidelines for the interpretation of variants.^48^ Yet clinical laboratories also merit policy attention. The vast majority of submissions to ClinVar are from clinical rather than research laboratories.^49^ The companion paper from Deverka and colleagues makes an important contribution to articulating the why and how of payer participation in building the commons.^50^ If, as our panelists suggest, the interest of payers in the data-sharing enterprise is not clear and simple, it is important to flesh out the business case for payers to join other stakeholders in providing incentives for clinical laboratories to share data.

Our findings have several limitations. First, although the goal of a policy Delphi is not consensus, our panelists did not converge on many policy options. While that might make implementing any specific policy option challenging, the strengths of a policy Delphi are in the depth and breadth of points to consider as policy options are deliberated by policy makers. Second, the data are limited by the experts who participated. While the Delphi panel was carefully constructed to include diverse opinions from individuals who have direct experience with cancer genomics, not all perspectives were captured, for example, funders, journal editors, and policy makers. We mitigated the effects of this limitation by supplementing the Delphi process through interviews with individuals outside of the Delphi panel^51^ to inform the final survey. Additionally, the nature of a Delphi process requires a small sample size.^52^ However, two Delphi panelists were unable to complete the final round, which may have impacted our findings. Despite these limitations, our findings provide important insights into effective and feasible policy options that could further enable cancer variant commons.

## V. CONCLUSION

Outcomes from the final round of our modified policy Delphi process revealed that the most feasible, effective, and high-priority policy options to advance the genomic cancer variant commons were connected to funders. The policy options included establishing stable funding for data-sharing initiatives, advancing equity, implementing data quality standards, strengthening incentives for data sharing, and protecting privacy and data security. These prioritized policy options conflict with panelists’ perceptions about the sustainability of funding for data sharing. Cultural shifts to enable stable funding for data-sharing initiatives were noted as critical to long-term success. Equity continues to be a high priority domain to address, but a clear path forward remains elusive. Data resources are important actors, but panelists were unconvinced they could resolve the identified issues alone. Policy makers should consider these findings, and especially the points to consider raised by Delphi panelists, to ensure policies are implemented that facilitate sustainable cancer variant data commons that provide quality data, protect privacy, promote equity, and improve cancer care.

## FUNDING

Research reported in this publication was supported by the National Cancer Institute of the National Institutes of Health under Award Number R01CA237118 (PIs: Robert Cook-Deegan and Amy McGuire). The content is solely the responsibility of the authors and does not necessarily represent the official views of the National Institutes of Health.

## AUTHORS’ CONTRIBUTIONS

Funding acquisition: BCD, ALM; conceptualization, methodology, and supervision: JOR, MAM, BCD, ALM; data analysis, visualization, writing (original draft): JOR, AD, JG, MAM; writing-review and editing: JOR, AD, JG, VR, JB, CG, MAM, BCD, ALM.

## ETHICS DECLARATION

The Baylor College of Medicine Institutional Review Board (IRB) approved this study. All participants provided consent as approved by the IRB.

Sulston: Outcomes of a modified policy Delphi

## Supplementary Material

Sulston_DelphiSurvey4_Supplement_RRFinal_lsad022

## Footnotes

1 Nat’l Insts. of Health Off. of Sci. Pol’y, *NIH Genomic Data Sharing*, NIH.gov (Aug. 2014), https://grants.nih.gov/grants/guide/notice-files/not-od-14-124.html (https://perma.cc/U6TE-632P); Nat’l Cancer Inst., *Cancer Moonshot^SM^ Public Access and Data Sharing Policy*, Cancer.gov (updated Dec. 16, 2021), https://www.cancer.gov/research/key-initiatives/moonshot-cancer-initiative/funding/public-access-policy (https://perma.cc/F6XP-KBGR).

2 Nat’l Cancer Inst., *Genomic Data Commons*, Cancer.gov, https://gdc.cancer.gov/ (accessed Jan. 11, 2023).

3 Robert Grossman, *How Data Commons Can Support Open Science*, The University of Chicago Center for Translational Data Science (2019), https://ctds.uchicago.edu/news/2019/5/2/how-data-commons-can-support-open-science (accessed Apr. 17, 2023); Janis Geary et al., *Conceptual, Legal, and Ethical Considerations for Stakeholders*, in Bioinformatics, Medical Informatics and the Law 12–30 (Jorge L. Contreras, A. J. Cuticchia, G. J. Kirsch eds., 2022).

4 Janis Geary et al., *Development of an Open Database of Genes Included in Hereditary Cancer Genetic Testing Panels Available From Major Sources in the US*, JAMA Oncol. 1 (Feb. 3, 2022).

5 Amanda E. Toland et al., *Lessons Learned from Two Decades of* BRCA1 *and* BRCA2 *Genetic Testing: The Evolution of Data Sharing and Variant Classification*, 21 Genet. Med. 1476 (2019).

6 Clay McLeod et al., *St. Jude Cloud: A Pediatric Cancer Genomic Data-Sharing Ecosystem*, 11 Cancer Discovery 1082 (2021).

7 Ricky Erway & Amanda Rinehart, *If You Build It, Will They Fund? Making Research Data Management Sustainable*, OCLC Res. (Jan. 2016), https://www.oclc.org/content/dam/research/publications/2016/oclcresearch-making-research-data-management-sustainable-2016.pdf (https://perma.cc/P4VV-UR6G); Nat’l Insts. of Health Off. of Sci. Pol’y, *NIH Strategic Plan for Data Science* 5 (June 2018), https://datascience.nih.gov/sites/default/files/NIH_Strategic_Plan_for_Data_Science_Final_508.pdf (https://perma.cc/ZUG2-RQN5).

8 Alex H. Wagner et al., *A Harmonized Meta-Knowledgebase of Clinical Interpretations of Somatic Genomic Variants in Cancer*, 52 Nat. Genet. 448 (2020).

9 Kilannin Krysiak et al., *A Community Approach to the Cancer-Variant-Interpretation Bottleneck*, 3 Nat. Cancer 522 (2022).

10 Kenneth D. Doig et al., *Findings from Precision Oncology in the Clinic: Rare, Novel Variants are a Significant Contributor to Scaling Molecular Diagnostics*, BMC Med. Genomics, 70 (Mar. 26, 2022).

11
*Id.*

12
*Id.,* at 8.

13 Geary et al., *supra* note 4; Janis Geary et al., *Equity and Inclusion in Hereditary Cancer Resources,* J. Law Biosci. (in review).

14 Marc Fiume et al., *Federated Discovery and Sharing of Genomic Data using Beacons*, 37 Nat. Biotechnol. 220 (2019).

15 James Brian Byrd et al., *Responsible, Practical Genomic Data Sharing that Accelerates Research*, 21 Nat. Rev. Genet. 615 (2020).

16 Gamze Gürsoy et al., *Functional Genomics Data: Privacy Risk Assessment and Technological Mitigation*, 23 Nat. Rev. Genet. 245 (2022).

17 Mary A. Majumder et al., *Beyond Our Borders? Public Resistance to Global Genomic Data Sharing*, PLOS Biol. 1 (Nov. 2, 2016).

18 Anna Hermansen, Dean A. Regier & Samantha Pollard, *Developing Data Sharing Models for Health Research with Real-World Data: A Scoping Review of Patient and Public Preferences*, 46 J. Med. Syst. 86 (2022); Nanibaa’ A. Garrison et al., *A Systematic Literature Review of Individuals’ Perspectives on Broad Consent and Data Sharing in the United States*, 18 Genet. Med. 663 (2016); Anna Middleton et al., *Global Public Perceptions of Genomic Data Sharing: What Shapes the Willingness to Donate DNA and Health Data?*, 107 Am. J. Hum. Genet. 743 (2020).

19 Vasiliki Rahimzadeh et al., *Leveraging Algorithms to Improve Decision-Making Workflows for Genomic Data Access and Management*, Biopreserv Biobank. 1 (June 30, 2022).

20 Majumder et al., *supra* note 17; Weiyi Xia et al., *Enabling Realistic Health Data Re-Identification Risk Assessment through Adversarial Modeling*, 28 J. Am. Med. Inform. Assoc. 744 (2021).

21 Katrina Learned et al., *Barriers to Accessing Public Cancer Genomic Data*, 6 Sci. Data 98 (2019).

22 Mary A. Majumder et al., *Challenges to Building a Gene Variant Commons to Assess Hereditary Cancer Risk: Results of a Modified Policy Delphi Panel Deliberation*, J. Personalized Med. 1 (July 8, 2021).

23 Juli M. Bollinger et al., *BRCA1/2 Variant Data-Sharing Practices*, 47 J. Law. Med. Ethics 88 (2019); Majumder et al., *supra* note 17.

24
Kathryn Fitch et al., The RAND/UCLA Appropriateness Method User’s Manual (2001), https://www.rand.org/pubs/monograph_reports/MR1269.html.

25 Majumder et al., *supra* note 22.

26 Interview participant characteristics are reported in Christi J. Guerrini et al., *Fresh Takes on Five Health Data Sharing Domains: Quality, Privacy, Equity, Incentives, and Sustainability*, 6 Front. Big Data 1095119 (2023).

27 Majumder et al., *supra* note 22.

28
Harold A. Linstone & Murray Turoff, The Delphi Method: Techniques and Applications 618 (1975); Robert C. de Loe, *Exploring Complex Policy Questions Using the Policy Delphi: A Multi-Round, Interactive Survey Method*, 15 Appl. Geog. 53 (1995).

29 One policy option in the equity domain [E3: ‘Funders could create mechanisms that prioritize research on hereditary cancer involving diverse participants and research teams (eg having diverse research teams to help achieve fuller understanding and build trust with diverse communities)’] was inadvertently omitted from the rating section of the initial distribution of the survey, and only four participants scored it for effectiveness and feasibility. We multiplied the score from these four participants by 5.5 to make the score comparable to other options in the domain. All panelists were provided the option for the ranking section, however, and it only ranked eighth in terms of priority within the equity domain.

30
Linstone & Turoff, *supra* note 28; de Loe, *supra* note 28.

31 de Loe, *supra* note 28.

32 In the Round 4 survey, we identified the National Institutes of Health (NIH) and private foundations as examples for ‘funders.’

33
*News*: *NHGRI Anvil Cloud Platform Now Supports Free Export of GTEx Data*, GTEx Portal (Nov. 20, 2020), https://gtexportal.org/home/home/news?id=360 (perma.cc/MW44-2R6D).

34 Bollinger et al., *supra* note 23.

35 Nat’l Insts. of Health Off. of Sci. Pol’y, *NIH Strategic Plan for Data Science*, *supra* note 6, at 5 (emphasis in original).

36 Chief Ben-Eghan et al., *Don’t Ignore Genetic Data from Minority Populations*, 585 Nature 184 (2020).

37
Committee on the Use of Race, Ethnicity, and Ancestry as Population Descriptors in Genomics Research et al., Using Population Descriptors in Genetics and Genomics Research: A New Framework for an Evolving Field (2023), www.ncbi.nlm. http://nih.gov/books/NBK589855/ (accessed Apr. 17, 2023).

38 Geary et al., *Equity and Inclusion*, *supra* note 13.

39 Heidi L. Rehm et al., *GA4GH: International Policies and Standards for Data Sharing Across Genomic Research and Healthcare*, Cell Genom. 1 (Nov. 10, 2021); James Casaletto et al., *Federated Analysis of* BRCA1 *and* BRCA2 *Variation in a Japanese Cohort*, Cell Genom. 1 (Mar. 9, 2022).

40 Ida Sim et al., *Time for NIH to Lead on Data Sharing*, 367 Science 1308 (2020).

41 Nat’l Insts. of Health, NOT-OD-21-013: Final NIH Policy for Data Management and Sharing, NIH.gov (released Oct. 29, 2020), https://grants.nih.gov/grants/guide/notice-files/NOT-OD-21-013.html (https://perma.cc/6LEM-3477).

42 Tammy M. Frisby & Jorge L. Contreras, *The National Cancer Institute Cancer Moonshot Public Access and Data Sharing Policy—Initial Assessment and Implications*, Data Policy 2 (Jul. 2, 2020).

43 The Genomic Data Sci. Cmty. Network, *Diversifying the Genomic Data Science Research Community*, 32 Genome Rsch. 1231 (2022)

44
*IndigiData - Indigenous Data Science Education*, NativeBio.org (2022), https://indigidata.nativebio.org/ (https://perma.cc/N49X-WC7Q).

45 David B. Resnik et al., *Effect of Impact Factor and Discipline on Journal Data Sharing Policies*, 26 Accountability Rsch. 139 (2019); Florian Naudet et al., *Medical Journal Requirements for Clinical Trial Data Sharing: Ripe for Improvement*, PLOS Med. 1 (Oct. 25, 2021).

46 Naudet et al., *supra* note 45. Naudet and colleagues focus on sharing of de-identified or anonymized data from clinical trials and note that legal constraints in the European Union and elsewhere may affect data sharing. Policies recently implemented in the US recognize that sharing under controlled access conditions may be the most appropriate option for human data that is identifiable or considered sensitive for other reasons (absent explicit informed consent to open access conditions). Nat’l Insts. of Health, *supra* note 44; Nat’l Insts. of Health, Supplemental Information to the NIH Policy for Data Management and Sharing: Protecting Privacy When Sharing Human Research Participant Data, https://grants.nih.gov/grants/guide/notice-files/NOT-OD-22-213.html (released September 21, 2022).

47 Gregory S. Barsh et al., *PLOS Genetics Data Sharing Policy: In Pursuit of Functional Utility*, PLOS Genetics 1 (Dec. 14, 2015).

48 Sue Richards et al., *Standards and Guidelines for the Interpretation of Sequence Variants: A Joint Consensus Recommendation of the American College of Medical Genetics and Genomics and the Association for Molecular Pathology*, 17 Genet Med. 405 (2015).

49 Geary et al., *supra* note 4.

50 Pat Deverka et al., *Coding, Coverage, and Reimbursement for Genetic Panel Tests of Inherited Cancer Risk,* J. Law Biosci. (forthcoming).

51 Guerrini et al., *supra* note 26.

52
Linstone & Turoff, *supra* note 28.

