# Supplementary material for: Policy options to facilitate cancer genomic variant data sharing: outcomes of a modified policy Delphi
Source: J Law Biosci. 2023 Jul 14;10(2):lsad022. doi: 10.1093/jlb/lsad022 (PMC10347303; doi:10.1093/jlb/lsad022)
Supplement: Sulston_DelphiSurvey4_Supplement_RRFinal_lsad022 [file sulston_delphisurvey4_supplement_rrfinal_lsad022.docx]

**Supplement Tables and Figures**

Supplement Table 1: Summary of results for each policy option, organized by highest to lowest total score within domains

| **Domain** | | **Statement** | **Policy Option Label** | **Effectiveness Score** | **Feasibility Score** | **Counts of Don’t Knows** | **Priority Rank Score** | **Priority Rank Ratio** | **Total Score** | **Actor^†^** |
| --- | --- | --- | --- | --- | --- | --- | --- | --- | --- | --- |
| Financial Sustainability | | Funders could provide stable funding for a trusted entity (e.g., GA4GH). | FS1 | 31 | 21 | 1 Effective  2 Feasible | 34 | 1.8 | 86 | Funders |
|  |  | Funders could focus on broadening use of existing data resources (e.g., ClinVar). | FS7 | 26 | 28 | 2 Effective  2 Feasible | 18 | 1.0 | 72 | Funders |
|  |  | Funders could invest in low-cost data archiving/storage. | FS4 | 17 | 26 | 1 Effective  2 Feasible | 22 | 1.2 | 65 | Funders |
|  |  | Funders could develop metrics to guide decisions about funding for data resources | FS2 | 21 | 21 | 5 Effective  4 Feasible | 16 | 0.8 | 58 | Funders |
|  |  | Funders could develop funding mechanisms to merge existing data resources. | FS3 | 19 | 14 | 3 Effective  6 Feasible | 18 | 1.0 | 51 | Funders |
|  |  | Public-private partnerships could give private financial supporters preferential access to some data and tools. | FS5 | 3 | 4 | 2 Effective  2 Feasible | 10 | 0.5 | 17 | Public-private partnerships |
|  |  | Data resources could be privately financed using a fee-for-service payment model (i.e., “pay-to-play” or charging to access data). | FS6 | -4 | 4 | 1 Effective  2 Feasible | 10 | 0.5 | 10 | Data Resources |
| Equity | | Funders could create funding mechanisms to support studies with small samples if from underrepresented populations. | E6 | 24 | 25 | 3 Effective  2 Feasible | 12 | 1.1 | 61 | Funders |
|  |  | Data resources could equip lower-resourced (often minority-serving) institutions and communities to utilize their data to conduct research. | E8 | 26 | 16 | 1 Effective  1 Feasible | 13 | 1.2 | 55 | Data Resources |
|  |  | Funders could conduct community needs assessments to identify community priorities and create greater alignment between funding and those priorities. | E1 | 19 | 18 | 1 Effective  2 Feasible | 15 | 1.4 | 52 | Funders |
|  |  | Journals could require transparency about dataset diversity and provenance and/or how findings relate to health equity. | E9 | 16 | 17 | 2 Effective  1 Feasible | 16 | 1.5 | 49 | Journals |
|  |  | Funders could create mechanisms that prioritize research on hereditary cancer involving diverse participants and research teams (e.g., having diverse research teams to help achieve fuller understanding and build trust with diverse communities). | E3 | 22 | 14 | 1 Effective  0 Feasible | 10 | 0.9 | 46 | Funders |
|  |  | Data resources could develop and implement creative and inclusive approaches to governance (e.g., multidisciplinary oversight including patient advocates, which could involve adopting strategies developed by All of Us and PCORI). | E7 | 17 | 16 | 2 Effective  3 Feasible | 13 | 1.2 | 46 | Data Resources |
|  |  | Data resources and researchers could use multidisciplinary approaches to engagement, including involving social scientists. | E11 | 20 | 19 | 1 Effective  1 Feasible | 5 | 0.5 | 44 | Data Resources |
|  |  | Funders could promote incorporating community engagement in research training and continuing education. | E4 | 16 | 17 | 1 Effective  0 Feasible | 9 | 0.8 | 42 | Funders |
|  |  | Funders could create funding mechanisms to support long-term community relationship building. | E5 | 16 | 9 | 2 Effective  1 Feasible | 10 | 0.9 | 35 | Funders |
|  |  | Data resources and researchers could compensate and/or give back to underrepresented communities (e.g., funders could encourage or require them to build a community benefit plan into their grant applications). | E12 | 13 | 7 | 1 Effective  1 Feasible | 13 | 1.2 | 33 | Data Resources |
|  |  | Data resources and researchers could address the potential for community harm and stigma (e.g., adopting or building on All of Us policies and processes focused on avoiding stigmatizing research areas). | E10 | 9 | 13 | 4 Effective  5 Feasible | 5 | 0.5 | 27 | Data Resources |
|  |  | Funders could require the data resources to conduct and publish health equity audits. | E2 | 7 | 7 | 4 Effective  4 Feasible | 7 | 0.6 | 21 | Funders |
| Data Quality | | Funders could fund data sharing infrastructure (e.g., setting standards, data cleaning, curation). | Q2 | 35 | 28 | 2 Effective  2 Feasible | 37 | 3.6 | 100 | Funders |
|  |  | Data resources could include rigorous quality checks in data selection and curation processes (e.g., gnomAD) and/or attach quality ratings assessed by standard metrics to data (e.g., ClinVar). | Q8 | 24 | 17 | 3 Effective  3 Feasible | 18 | 1.8 | 59 | Data Resources |
|  |  | Funders could incentivize data contributors to comply with standards (i.e., preferred access or funding access to the data resources). | Q1 | 25 | 19 | 3 Effective  3 Feasible | 12 | 1.2 | 56 | Funders |
|  |  | Professional societies could develop data standards. | Q12 | 24 | 22 | 3 Effective  2 Feasible | 9 | 0.9 | 55 | Professional societies |
|  |  | Core data elements of data resources could be clearly defined to set standards. | Q4 | 26 | 21 | 5 Effective  5 Feasible | 7 | 0.7 | 54 | Broad actors |
|  |  | Data resources could create a clear and easy-to-follow method to standardize data collection and characterization. | Q10 | 24 | 14 | 2 Effective  2 Feasible | 11 | 1.1 | 49 | Data Resources |
|  |  | Funders could fund the development of technology to link patient data from many sources (e.g., labs, hospitals, clinical settings). | Q3 | 24 | 10 | 2 Effective  4 Feasible | 8 | 0.8 | 42 | Funders |
|  |  | Data resources could adopt a "silver standard" instead of a “gold standard” for data quality while being transparent about imperfections. | Q7 | 16 | 18 | 3 Effective  4 Feasible | 6 | 0.6 | 40 | Data Resources |
|  |  | A central body could be identified to harmonize and enforce standards for data quality. | Q5 | 17 | 9 | 4 Effective  5 Feasible | 8 | 0.8 | 34 | Central Body |
|  |  | Data resources could produce a multi-user, friendly, API-based data contribution platform to improve quality. | Q6 | 12 | 14 | 8 Effective  8 Feasible | 1 | 0.1 | 27 | Data Resources |
|  |  | Individual researchers, institutions, clinical labs, and research groups could provide “assertion criteria” (i.e., interpretation standards) in addition to sharing the data pedigree, protocols, and workflow. | Q13 | 10 | 7 | 9 Effective  9 Feasible | 3 | 0.3 | 20 | Broad actors |
|  |  | Data resources could implement artificial intelligence (AI) approaches to clean, extrapolate, and interpret the clinical significance of variant data. | Q9 | 4 | -4 | 6 Effective  7 Feasible | 0 | 0.0 | 0 | Data Resources |
|  |  | Data resources could crowdsource data characterization while also providing attribution for deposited data, allowing speculative data to exist. | Q11 | -4 | -4 | 7 Effective  7 Feasible | 0 | 0.0 | -8 | Data Resources |
| Incentives | | Journals could condition publication on submission of sufficient data for replication. | I9 | 26 | 23 | 0 Effective  0 Feasible | 19 | 1.3 | 68 | Journals |
|  |  | Funders could require that data-sharing plans be peer-reviewed for grant selection. | I4 | 16 | 27 | 2 Effective  0 Feasible | 19 | 1.3 | 62 | Funders |
|  |  | Funders could monitor funded projects and withhold funds or future grants from applicants who do not comply with approved data sharing plans. | I5 | 20 | 10 | 0 Effective  0 Feasible | 20 | 1.4 | 50 | Funders |
|  |  | Health insurers could exclude clinical labs from preferred networks if they don't share. | I1 | 20 | 8 | 2 Effective  4 Feasible | 18 | 1.2 | 46 | Health Insurer |
|  |  | Proficiency testing programs could use data sharing as one metric of lab proficiency. | I8 | 15 | 11 | 5 Effective  5 Feasible | 13 | 0.9 | 39 | Proficiency testing program |
|  |  | Health insurers could reward labs that share data with higher payments. | I3 | 13 | 4 | 2 Effective  5 Feasible | 9 | 0.6 | 26 | Health Insurer |
|  |  | Health insurers could penalize reimbursement up to 100% if labs don't share. | I2 | 13 | -2 | 4 Effective  6 Feasible | 14 | 1.0 | 25 | Health Insurer |
|  |  | Data resources could provide users submitting their data with financial and/or nonfinancial compensation (e.g., co-authorship of papers, direct engagement with standard setting). | I7 | 2 | 5 | 3 Effective  4 Feasible | 9 | 0.6 | 16 | Data Resources |
|  |  | Data resources could provide tiered access to data and interpretation tools based on data contributions (i.e., the more you share, the more you can access). | I6 | 0 | 1 | 2 Effective  2 Feasible | 9 | 0.6 | 10 | Data Resources |
| Privacy and Security | | Funders, clinical labs, individual researchers, institutions, and end users of data could adopt federated models of data sharing to avoid having a centralized database, where data are uploaded and downloaded locally, which would minimize risks of re-identification and reduce harms from security breaches. | PS10 | 24 | 22 | 4 Effective  6 Feasible | 26 | 2.2 | 72 | Broad actors |
|  |  | Funders, institutions, and end users of data could invest in the development and use of novel technologies geared towards protecting privacy and enhancing data security (e.g., leveraging synthetic data to reduce re-identification risk, and leveraging secure computational methods to allow analysis of data without moving data). | PS9 | 23 | 22 | 2 Effective  4 Feasible | 23 | 1.9 | 68 | Broad actors |
|  |  | Data resources, institutions, and individual researchers could be more transparent about security risks and potential harms. | PS8 | 14 | 15 | 2 Effective  2 Feasible | 13 | 1.1 | 42 | Data Resources |
|  |  | Companies who generate and store data, institutions, or the National Academies could help develop best practices for privacy and security of data. | PS11 | 20 | 16 | 1 Effective  1 Feasible | 5 | 0.4 | 41 | Companies |
|  |  | Congress could expand GINA by prohibiting discriminatory use of data by long-term care, disability, and life insurers. | PS2 | 23 | -1 | 3 Effective  2 Feasible | 17 | 1.4 | 39 | Congress |
|  |  | Funders could conduct or fund research on privacy and security mitigation/management strategies and associated costs. | PS6 | 14 | 15 | 4 Effective  3 Feasible | 10 | 0.8 | 39 | Funders |
|  |  | Data resources could conduct regular risk assessments (e.g., every 6 months) that include review of changes, risk threats, technical protections, and relevant laws and policies. | PS4 | 10 | 8 | 2 Effective  2 Feasible | 9 | 0.8 | 27 | Data Resources |
|  |  | Ethics boards overseeing data resources could monitor use, and impose sanctions for violations. | PS7 | 10 | 4 | 3 Effective  3 Feasible | 12 | 1.0 | 26 | Ethics Boards |
|  |  | Congress could pass laws that address re-identification and related risks. | PS3 | 9 | -10 | 6 Effective  6 Feasible | 6 | 0.5 | 5 | Congress |
|  |  | Congress could strengthen HIPAA by expanding coverage of privacy protections, strengthening enforcement of privacy rules, and/or enhancing sanctions for privacy violations. | PS1 | 11 | -13 | 2 Effective  3 Feasible | 2 | 0.2 | 0 | Congress |
|  |  | Data resources could create a mechanism to compensate those whose privacy is violated by security breaches. | PS5 | 5 | -9 | 3 Effective  3 Feasible | 3 | 0.3 | -1 | Data Resources |
|  | ^†^Examples of actors are shown at the beginning of the survey provided in Supplementary materials. | | | | | | | | | |

Supplement Table 2: Average effectiveness and feasibility scores of policy options by domain

| **Domain** | **Average Effectiveness Score** | **Average Feasibility Score** |
| --- | --- | --- |
| Financial Sustainability | 16.1 | 16.9 |
| Equity | 17.1 | 14.8 |
| Data Quality | 18.2 | 13.2 |
| Incentives | 13.9 | 9.7 |
| Privacy and Security | 14.8 | 6.3 |

Supplement Figure 1: Financial sustainability domain effectiveness, feasibility, and priority rank scores

Supplement Figure 2: Equity domain effectiveness, feasibility, and priority rank scores

Supplement Figure 3: Data quality domain effectiveness, feasibility, and priority rank scores

Supplement Figure 4: Incentives domain effectiveness, feasibility, and priority rank scores

Supplement Figure 5: Privacy and security domain effectiveness, feasibility, and priority rank scores
